# Comment on ‘Time for hard choices – a new global order for mental health’: Why migrants should no longer be ignored

**DOI:** 10.1177/00207640221112322

**Published:** 2022-08-17

**Authors:** Grace Crowley

**Affiliations:** South London and Maudsley NHS Foundation Trust and the Institute of Psychiatry, Psychology and Neuroscience, London, UK

In calling for a ‘new global order for mental health’, Persaud and colleagues highlighted that only one of the six targets to be achieved by 2020 set out in the Comprehensive Mental Health Action Plan (MHAP) had been met (World Health Organization [WHO], 2021a). The Mental Health Atlas, which tracks the implementation of the MHAP, considers country-level data but does not capture the impact of geopolitical factors such as internal and external migration and their impact on mental health systems. It would appear that even less progress has been made towards achieving the targets among migrant groups, especially forced migrants and displaced populations, among whom there are high rates of mental illness (Blackmore et al., 2020) and who face additional barriers to accessing mental healthcare (Satinsky et al., 2019). It is important to emphasise why future iterations of the Mental Health Atlas should report data on migrants as well as specific subgroups of migrants particularly likely to face mental health difficulties.

Achieving a 10% reduction in the global suicide rate was the only MHAP target met by 2020. As this is a global average, we do not know whether suicide rates have reduced among migrant populations. Over three quarters of all suicides occur in low- and middle-income countries (WHO, 2021b), which are also host to the majority of the world’s refugees (United Nations High Commissioner for Refugees, 2021). Our understanding of how migration influences suicide rates is hindered because most countries do not routinely document migration status or even ethnicity on death records. Concerns about this have led to calls for coroners in the UK to report ethnicity and migration status (Cohen et al., 2020). It is entirely possible that low suicide rates may reflect inaccurate reporting, especially from countries where suicide is an illegal act. In addition, failing to consider migrants when presenting suicide rates in the context of global mental health perpetuates the misconception that suicide is universally a mental health issue. The rates of mental illness among those who die by suicide in low- and middle-income countries are lower than those seen in high-income countries (Knipe et al., 2019). Therefore, the extent to which mental illness determines suicide among migrants may be different to that of host populations. Monitoring of global suicide rates must be done alongside economic and social trends, and with an awareness that reduction in suicides may not necessarily reflect improvements in mental health care.

The proportion of member states that have implemented mental health policies, plans and laws in-keeping with human rights instruments remains disappointing and migrants are especially likely to be impacted by a lack of these safeguards. Experience of human rights abuses in their country of origin is associated with higher levels of psychological distress among migrants in the longer-term (Joly & Wheaton, 2020). Application of mental health laws also differentially affects migrants, for example, they are more likely to be compulsorily admitted to a psychiatric hospital compared to native groups (Barnett et al., 2019).

While global coverage of mental health services for people with psychosis (29%) and depression (40%) fell far short of the 80% MHAP target, migrant groups may face additional challenges to accessing services, such as language barriers, stigma and unfamiliarity with healthcare systems (Satinsky et al., 2019). Service coverage for migrants is therefore likely to be much lower than the figures reported in the MHAP.

Assessing mental health promotion and prevention strategies at the country-level risks undermining efforts to encourage the development of targeted interventions for different groups within society. For example, post-migration social-environmental factors such as living and working conditions, poverty and immigration systems are known to impact profoundly on the mental health of asylum seekers (Jannesari et al., 2020). Interventions that focus on a medical model of mental illness and ignore non-medical and non-psychological explanatory models are therefore likely to miss the mark.

Persaud and colleagues also discuss the impact of geopolitical factors on the landscape of mental health worldwide. Immigration continues to be a highly politicised topic in many countries; thus, the treatment of migrants cannot be disentangled from national politics and policies. Anti-immigration rhetoric created by those in power can directly impact on the mental health of migrants through the mediating effects of hate crime, discrimination and the perception of discrimination (which in itself has been shown to contribute to mental ill-health) but also indirectly, by shaping immigration control policies, and thus migrants’ rights and access to basic needs. The COVID-19 pandemic has highlighted stark inequalities in access to healthcare, and migrants have been disproportionately represented among COVID-19 cases and deaths (Hayward et al., 2021). Finally, climate change is a significant driver of both internal and international displacement. As time progresses, it will become increasingly important to study the interplay between climate change, displacement and mental health.

A more nuanced approach to data collection and reporting is required. Migration status should not be considered a luxury – a variable to present in demographic tables if data is available. It should be deemed essential for providing a clearer picture of how the MHAP targets are being achieved. For this to be possible, individual countries need to start embedding migration status into healthcare data systems and death registers, which requires significant time and investment in infrastructure. In the meantime, more use should be made of data-linkage methodologies, that is, mapping migrant mental health and mortality outcomes onto pre-existing datasets that include migration status.

Continuing to ignore the impact of migration on mental health outcomes will lead to misjudgement and miscalculation. Considering country-level data alone will not capture mobile and transient populations, who may face assaults to mental health such as conflict and natural disasters and extreme difficulties accessing basic needs and mental health support. A ‘new global order’ for mental health must consider the increasingly globalised world in which we live on the one hand and increasing nationalistic and xenophobic rhetoric on the other. We have a moral responsibility to not only investigate the mental health outcomes of migrants and how countries are addressing their needs but to also evaluate global mental health trends in a joined-up manner.

## References

[bibr1-00207640221112322] Barnett P. Mackay E. Matthews H. Gate R. Greenwood H. Ariyo K. Bhui K. Halvorsrud K. Pilling S. Smith S. (2019, April). Ethnic variations in compulsory detention under the Mental Health Act: A systematic review and meta-analysis of international data. Lancet Psychiatry, 6(4), 305–317. 10.1016/s2215-0366(19)30027-6 30846354 PMC6494977

[bibr2-00207640221112322] Blackmore R. Boyle J. A. Fazel M. Ranasinha S. Gray K. M. Fitzgerald G. Misso M. Gibson-Helm M. (2020). The prevalence of mental illness in refugees and asylum seekers: A systematic review and meta-analysis. PLOS Medicine, 17(9), e1003337. 10.1371/journal.pmed.1003337 PMC7505461 32956381

[bibr3-00207640221112322] Cohen J. Katona C. Bhugra D. (2020). National data on suicide must include ethnicity. BMJ, 371, m4105. 10.1136/bmj.m4105 33109680

[bibr4-00207640221112322] Hayward S. E. Deal A. Cheng C. Crawshaw A. Orcutt M. Vandrevala T. F. Norredam M. Carballo M. Ciftci Y. Requena-Méndez A. Greenaway C. Carter J. Knights F. Mehrotra A. Seedat F. Bozorgmehr K. Veizis A. Campos-Matos I. Wurie F. McKee M. Kumar B. Hargreaves S. (2021). Clinical outcomes and risk factors for COVID-19 among migrant populations in high-income countries: A systematic review. Journal of Migration and Health, 3, 100041. 10.1016/j.jmh.2021.100041 33903857 PMC8061095

[bibr5-00207640221112322] Jannesari S. Hatch S. Prina M. Oram S. (2020, October). Post-migration social-environmental factors associated with mental health problems among asylum seekers: A systematic review. Journal of Immigrant and Minority Health, 22(5), 1055–1064. 10.1007/s10903-020-01025-2 32430778 PMC7441054

[bibr6-00207640221112322] Joly M.-P. Wheaton B. (2020, August, 1). Human rights in countries of origin and the mental health of migrants to Canada. SSM - Population Health, 11, 100571. 10.1016/j.ssmph.2020.100571 32322655 PMC7160446

[bibr7-00207640221112322] Knipe D. Williams A. J. Hannam-Swain S. Upton S. Brown K. Bandara P. Chang S. S. Kapur N. (2019, October). Psychiatric morbidity and suicidal behaviour in low- and middle-income countries: A systematic review and meta-analysis. PLOS Medicine, 16(10), e1002905. 10.1371/journal.pmed.1002905 PMC6785653 31597983

[bibr8-00207640221112322] Satinsky E. Fuhr D. C. Woodward A. Sondorp E. Roberts B. (2019, September). Mental health care utilisation and access among refugees and asylum seekers in Europe: A systematic review. Health Policy, 123(9), 851–863. 10.1016/j.healthpol.2019.02.007 30850148

[bibr9-00207640221112322] United Nations High Commissioner for Refugees. (2021). Refugee data finder. Retrieved February 16, 2021, from https://www.unhcr.org/refugee-statistics/

[bibr10-00207640221112322] World Health Organization. (2021a). Mental Health Atlas 2020. Author. https://www.who.int/publications/i/item/9789240036703

[bibr11-00207640221112322] World Health Organization. (2021b). Suicide worldwide in 2019: Golbal health estimates. Author. https://www.who.int/publications/i/item/9789240026643

